# The PD1 Molecule May Contribute to Lower Treatment-Free Remission Rates in Patients with Chronic Myeloid Leukemia with the e13a2 Transcript

**DOI:** 10.3390/jcm14072304

**Published:** 2025-03-27

**Authors:** Paulina Kwaśnik, Michał Kiełbus, Joanna Zaleska, Dorota Link-Lenczowska, Magdalena Zawada, Hubert Wysogląd, Tomasz Sacha, Krzysztof Giannopoulos

**Affiliations:** 1 Department of Experimental Hematooncology, Medical University of Lublin, 20-093 Lublin, Poland; 2 Diagnostic Department of Hematology and Genetics, The University Hospital in Kraków, 30-688 Kraków, Poland; 3 Department of Hematology, University Hospital in Kraków, 30-688 Kraków, Poland; 4 Department of Hematology, Jagiellonian University Medical College in Kraków, 31-008 Kraków, Poland

**Keywords:** chronic myeloid leukemia, treatment-free remission, imatinib withdrawal, transcript type, immune biomarker

## Abstract

**Background/Objectives**: Chronic myeloid leukemia (CML) is characterized by the presence of the *BCR::ABL1* fusion gene, most commonly in the e14a2 or e13a2 variants. Studies show that the transcript type in CML may be important for achieving treatment-free remission (TFR). This study aimed to immunologically characterize CML patients with e13a2 and e14a2 transcripts to search for differences that may contribute to achieving remission in patients after therapy withdrawal. **Methods**: Using multicolor flow cytometry, we analyzed the differences in the immune system at the time of imatinib discontinuation and the early stage of TFR in fifty-one CML patients with different transcripts. RQ-PCR and ddPCR were used to monitor the dynamics of *BCR::ABL1* transcript changes. The patients were grouped using principal component analysis (PCA) based on the percentage of detected immune cells that were classified as populations consistently selected by the MCFS-ID algorithm from randomly selected data. **Results**: PCA separated CML patients into two groups defined by k-means clustering, indicating significant heterogeneity within the studied population. We found a significant association between Cluster metrics (Cluster 1 and 2) and *BCR::ABL1* transcript types (e13a2 or e14a2) (*p* = 0.003, 95% CI: 0.026–0.595, OR = 0.14, Fisher test). The e13a2 transcript was less frequent in Cluster 2 than in Cluster 1, while e14a2 was more common in Cluster 2. Additionally, patients grouped into Cluster 1 had significantly higher percentages of the PD1 expressing populations cDC PD1^+^, CD56^dim^CD16^+^PD1^+^, CD8^+^PD1^+^, CD4^+^PD1^+^, and CD19^+^PD1^+^, as identified by the MCFS-ID algorithm, compared to patients in Cluster 2. **Conclusions**: Our results suggest that immunological differences may be related to the *BCR::ABL1* transcript type, which could affect the number of active CML cells represented by the *BCR::ABL1* transcript amount and thus may determine molecular recurrence.

## 1. Introduction

Chronic myeloid leukemia (CML) is a myeloproliferative neoplasm with a presenting incidence rate of 2 cases per 100,000, accounting for approximately 15% of all de novo adult leukemias diagnosed yearly [1].

Since the introduction of TKIs, the survival rates in CML patients have improved significantly. TKIs provide a life expectancy almost comparable to that of healthy individuals, therefore, CML is no longer a fatal disease but a chronic condition with comorbidities that arise with age. However, despite the clear clinical benefits associated with the use of TKIs, prolonged treatment duration contributes to the occurrence of adverse effects in many patients, which can significantly reduce their quality of life. Furthermore, from a pharmacoeconomic point of view, prolonged treatment duration generates high treatment costs. Moreover, some patients experience treatment failure or resistance. The development of mutations in the BCR:ABL1 kinase domain, often as a result of long-term treatment, leads to the development of resistance to previously used treatments, which requires the consideration of more potent TKIs, which are often associated with greater toxicity.

One of the current goals of CML is to achieve long-term treatment-free remission (TFR) [2]. To date, clinical trials of tyrosine kinase inhibitor (TKI) discontinuation in CML have shown that approximately 40–50% of patients remain in remission despite treatment discontinuation [3,4,5,6,7]. However, some patients experience a molecular recurrence for unexplained reasons, most often in the first months after discontinuing treatment. Some earlier studies have suggested that maintaining remission may be the result of restoring immune surveillance in CML [8,9,10,11,12]. Our previous results proved that changes in the percentages of immune cell populations already occur at the early stage of TFR [13]. Worldwide the research is ongoing in order to understand the mechanisms explaining the phenomenon of maintaining remission only in some patients after stopping treatment [14,15,16,17].

CML is characterized by the presence of the *BCR::ABL1* fusion gene, which most commonly exists as the e14a2 or e13a2 variants. Studies show that the transcript type in CML may be important for maintaining TFR [18,19]. Some publications suggest that the e14a2 transcript is associated with a faster and deeper molecular response, more favorable responses to treatment, and better long-term outcomes such as overall survival, progression-free survival, and failure-free survival [20,21,22,23].

This study aimed to immunologically characterize CML patients with e13a2 and e14a2 transcript types to search for immunological differences that may contribute to maintaining at least major molecular remission (MMR) for the patient after stopping treatment.

## 2. Patients and Methods

### 2.1. Patients

Patients with CML in the chronic phase who were treated with imatinib and achieved a deep molecular response at the minimal level of molecular response MR4 with duration of r for least 2 years were included in this study. Peripheral blood samples of the patients were collected at the time of treatment discontinuation and at 3 months of TFR, both in patients with persistent remission and in those with molecular relapse of the disease. Molecular recurrence was defined as confirmed loss of MMR, assessed on two time points one month apart. The exclusion criteria from this study included the failure to achieve DMR during imatinib treatment, diagnosis of accelerated or blastic phase CML, and withdrawal of patient consent to participate in this study.

This study was approved by the Ethics Committee of the Medical University of Lublin (approval number KE-0254/174/2017), and informed consent was obtained under the Declaration of Helsinki. All patients provided written consent to participate in this study of the Polish Adult Leukemia Study Group.

### 2.2. Flow Cytometry for Detection of Immune Populations

Peripheral blood mononuclear cells (PBMC) were isolated using Biocoll (Biochrom, Berlin, Germany) density gradient centrifugation. Cell viability, expected to exceed 95%, was determined using trypan blue staining (Sigma Aldrich, Saint Louis, MO, USA). The viable cell counts were performed in a Neubauer chamber (Zeiss, Oberkochen, Germany). The cells were then stained with multicolor panels for cytometric analysis using a FACSLyric flow cytometer (Becton Dickinson, San Jose, CA, USA). To detect specific immune populations, the following fluorochrome-labeled monoclonal antibodies were used for extracellular staining:

- T cells, B cells, and regulatory T cells (Treg): anti-CD3-BV510 (catalog No: 564713), anti-CD4-PerCP (catalog No: 345770), anti-CD8-FITC (catalog No: 555634), anti-CD19-APC-Cy7 (catalog No: 557791), anti-CD25-PE-Cy7 (catalog No: 557741), and anti-CD127-BV421 (catalog No: 562436);
- Natural killer (NK) cells and natural killer T (NKT) cells: anti-CD45-BV510 (catalog No: 563204), anti-CD14-APC-H7 (catalog No: 560180), anti-CD3-PerCP (catalog No: 552851), anti-CD56-PE-Cy7 (catalog No: 557747), anti-CD16-BV421 (catalog No: 562874), anti-iNKT-FITC (catalog No: 558371), and anti-CD161-PE (catalog No: 556081);
- Conventional dendritic cells (cDC) and plasmacytoid dendritic cells (pDC): lineage cocktail FITC (anti-CD3, CD14, CD19, CD20, CD56; catalog No: 643397), anti-HLA-DR-BV510 (catalog No: 563083), anti-CD123-PE-Cy7 (catalog No: 560826), anti-BDCA-1-PE (catalog No: 564900), and anti-BDCA-2-BV421 (catalog No: 566427).

Furthermore, DC, NK, NKT, CD3^+^, CD4^+^, CD8^+^, and CD19^+^ cells were stained with the fluorochrome-labeled monoclonal antibody anti-PD1-APC (catalog No: 558694). To identify the threshold between PD1 positive and negative cells, FMO (fluorescence minus one) control staining was conducted. All antibodies were obtained from Becton Dickinson (San Jose, CA, USA). One million PBMC in each cytometric panel were incubated for 20 min in the dark at room temperature. For the DC, NK, and NKT assessments, the cells were washed twice with PBS. For the T cells, B cells, and Treg staining, the washing steps were performed using stain buffer (Becton Dickinson, San Jose, CA, USA). The Human FOXP3 Fix/Perm Buffer Set (Becton Dickinson, San Jose, CA, USA, catalog No: 560098) was used to detect regulatory T cells following the manufacturer’s protocol. Intracellular staining was performed with anti-FOXP3-PE (catalog No: 560046) monoclonal antibody for 30 min at room temperature. A minimum of 100,000 (300,000 for DC detection) stained cells from each sample were analyzed using flow cytometry. Data analyses were conducted using FACS Suite software (version 1.5, Becton Dickinson, San Jose, CA, USA).

### 2.3. Real-Time Quantitative Reverse Transcriptase Polymerase Chain Reaction (RQ-PCR) and Droplet Digital Polymerase Chain Reaction (ddPCR) for the Detection of BCR::ABL1

Total RNA was extracted according to the Chomczynski and Sacchi protocol with an assessment of the concentration and purity of isolated RNA using a NanoDrop Lite spectrophotometer (Thermo Fisher Scientific, Waltham, MA, USA). Reverse transcription reaction was performed according to the BIO-RAD protocol for the quantitative analysis of *BCR::ABL1* gene expression by RQ-PCR. The RQ-PCR method was carried out using the standards set by European Leukemia Net (ELN) and EUTOS for CML [24]. The ddPCR test was performed according to the described procedure [25].

### 2.4. Monte Carlo Feature Selection and Interdependency Discovery (MCFS-ID)

Monte Carlo feature selection (MCFS) was employed using the rmcfs package to identify the most informative features contributing to classification [26]. MCFS simplifies complex data while preserving key information. It identifies features crucial for classification by constructing decision trees from randomly selected feature–object subsets. Features are ranked based on their contribution, revealing both linear and non-linear relationships. Iterative ranking highlights the most impactful features, enabling the detection of synergistic effects in classification. The analysis involved conducting 100 iterations of MCFS on both a 70% subset of the dataset (training phase) and the full dataset (validation phase) to assess the stability of selected features. The algorithm was executed with mode = 1, cutoff permutations = 100, and cutoff method = ‘criticalAngle,’ while the other settings were maintained at their default values.

### 2.5. Principal Component Analysis (PCA)

The top five features consistently selected by the MCFS-ID algorithm from randomly sampled data were employed for PCA. We utilized the prcomp () function from the stats R package, with both the center and scale parameters set to TRUE, to perform the PCA. The results were then visualized using the factoextra R package. Visually, the scree plots were instrumental in determining the variance explained by different numbers of clusters, identifying the “elbow point.” This point is crucial, as it signifies where additional clusters do not substantially enhance the explanation of variance. Based on this analysis, using the k-means clustering method, it was determined that two clusters optimally minimize within-cluster variance while maximizing the distinction between clusters in the analyzed groups.

### 2.6. Statistical Analysis

We explored the association between the concentrations of the analyzed cell populations and the clinicopathological features of the disease. Continuous variables were assessed using the Mann–Whitney–Wilcoxon test, while categorical variables underwent analysis via either the asymptotic chi-square or Fisher’s test, based on the subgroup counts, as outlined in the stats package documentation. We addressed multiple comparison concerns by employing the Benjamini–Hochberg (BH) method to adjust *p* values and manage the false discovery rate. Statistically significant findings were those with an adjusted *p* value below 0.05. We conducted all data visualizations and statistical analyses using the ggpubr and ggplot2 packages within the R programming framework, version 4.1.3, and GraphPad Prism 8 Software.

## 3. Results

### 3.1. Characterization Summary of Enrolled Patients

This study analyzed fifty-one patients, including twenty-nine females (56.9%) and twenty-two males (43.1%), with a median age of 64 years (age range, 25–86 years), while the median age of the patients with the e13a2 transcript was lower than that of the patients with the e14a2 transcript (59.5 vs. 65, respectively). The observed cytopenias were mainly mild, with a difference between e13a2 and e14a2 transcript patients. A total of 50% of patients with the e13a2 transcript experienced a DMR loss at 3 months of TFR, while, in the group of patients with the e14a2 transcript, it was approximately 33%. There was no significant difference between the groups concerning age, gender, ELTS, EUTOS, Sokal, and Hasford scores, whereas a higher proportion of e13a2 patients was classified as the highest risk group for all scores used compared to the e14a2 patients. The clinical characteristics of the patients are summarized in detail in Table 1.

### 3.2. BCR::ABL1 Transcript Levels in CML Patients with the e13a2 or e14a2 Type in TFR

We analyzed *BCR::ABL1* transcript levels at imatinib discontinuation, at three months after withdrawal and at the time of molecular recurrence (the confirmed loss of MMR assessed on two occasions one month apart), using ddPCR as a technique relatively insensitive to differences in amplification kinetics.

**Figure 1:**
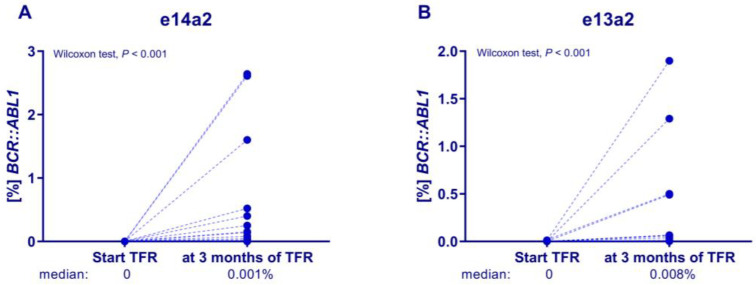
Analysis of the amount of *BCR::ABL1* transcript at the time of imatinib discontinuation compared to the amount in the third month of TFR depending on the transcript type. The *BCR::ABL1* levels were determined using the ddPCR method. The significance of differences in the dynamics of *BCR::ABL1* levels in patients with the e14a2 (**A**) and e13a2 (**B**) transcripts were assessed using the Wilcoxon test, displaying *p* values. Points on the graphs represent patients with the individual e14a2 (*N* = 33) and e13a2 (*N* = 18) transcripts.

The analyses of the dynamics of the *BCR::ABL1* level showed that the amount of the transcript already increased significantly in the 3rd month of TFR after discontinuation in both groups of patients with the e13a2 (*p* < 0.001, median 0 vs. 0.008%) or e14a2 (*p* < 0.001, median 0 vs. 0.001%) transcripts (Figure 1). Moreover, the median amounts of the e13a2 and e14a2 transcripts at 3 months of TFR were different, but statistical significance was not observed (*p* = 0.25, median 0.008 vs. 0.001%). Among the patients with sustained MMR, a trend towards lower *BCR::ABL1* levels was observed in the e14a2 group compared to that of the e13a2 group (*p* = 0.06, median 0.0005 vs. 0.0025%).

### 3.3. Selecting the Key Features That Distinguish Patients with e13a2 or e14a2 Transcripts

**Figure 2:**
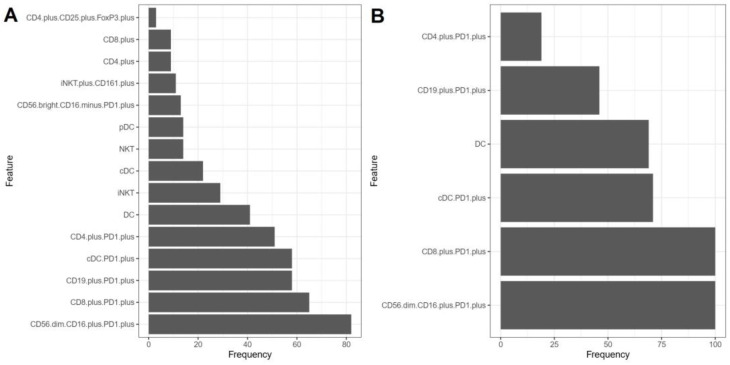
Feature selection analysis using the Monte Carlo feature selection and Interdependency Discovery (MCFS-ID) algorithm based on transcript types e13a2 and e14a2. (**A**) The frequency of selected features over one hundred permutations, each conducted on a randomly chosen subset comprising 70% of the total patient group. This plot illustrates how consistently certain features are identified as differentiators among patients when analyzed on a substantial but partial sample of the data. (**B**) The frequency of selected features across one hundred permutations conducted on the entire dataset. This plot provides insight into the robustness and stability of feature selection when the full scope of the data is considered, allowing for a comprehensive assessment of feature relevance.

In our study, we employed the Monte Carlo feature selection and Interdependency Discovery (MCFS-ID) algorithm to identify distinguishing features in patients based on the *BCR::ABL1* gene transcript type (e13a2 or e14a2). To verify the stability of the results, we conducted one hundred permutations on a randomly selected subset of patients, representing seventy percent of the total group. This subset served as a training dataset to ensure the robustness of our feature selection. Subsequently, to validate these findings, we performed an additional one hundred permutations on the entire dataset, enabling us to confirm the reproducibility and consistency of our results. Based on the results obtained from the MCFS-ID algorithm, we selected the following five features: percentages of CD56^dim^CD16^+^PD1^+^, CD8^+^PD1^+^, cDC PD1^+^, CD4^+^PD1^+^, and CD19^+^PD1^+^, which were most often identified as key during the one hundred permutations performed (Figure 2). An example result from one MCSF-ID analysis on the dataset is described in the Supplementary Materials, Figure S1.

### 3.4. Distinguishing Groups of Patients with e14a2 or e13a2 Transcripts Based on Their Principal Component Analysis Results

**Figure 3:**
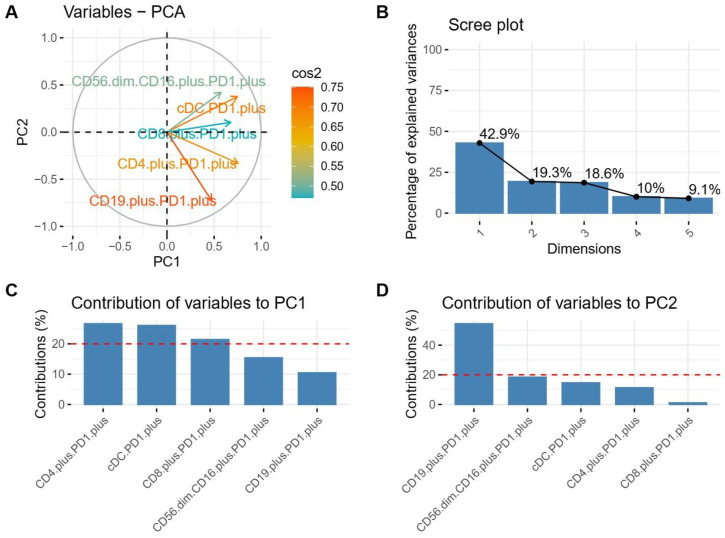
Determining critical cell populations for patient segmentation using principal component analysis (PCA). (**A**) PCA attributes. Arrow lengths represent the squared cosine values (cos^2^) for each variable, with higher values indicating a more substantial association with the respective principal components. (**B**) Scree plot of PCA. This plot illustrates the percentage of variance that each principal component (dimension) accounts for, highlighting the importance of each dimension. (**C**,**D**) Bar plots for the first and second principal components. The dotted line represents the mean variance explained by each PC (PC1 or PC2). Cell populations above the dotted line are most significant for that PC component variable. These plots detail how various variables contribute to the first (PC1) and second (PC2) principal components, respectively, offering insights into the dataset’s structure and pinpointing the compounds that significantly impact the variance explained by PC1 and PC2.

For additional validation and deeper analysis of the data structure, we decided to apply principal component analysis (PCA) on five selected populations, including CD56^dim^CD16^+^PD1^+^, CD8^+^PD1^+^, cDC PD1^+^, CD4^+^PD1^+^, and CD19^+^PD1^+^. The PCA revealed distinct clustering of CML patients, pointing towards substantial heterogeneity within the examined patient population. The first two principal components (PC1 and PC2) contributed the most to this separation and explained 62.2% of the variability (42.9% and 19.3%, respectively) (Figure 3A,B), suggesting that the variation in the percentages of the analyzed immunological cell populations primarily drove the observed clustering. Further investigation into the loading scores of the individual markers on the first two components identified specific cell populations contributing to the observed separation. The highest percentage of contribution of variables to the first principal component (Dim-1 or PC1) was seen for the CD4^+^PD1^+^, cDC PD1^+^, and CD8^+^PD1^+^ levels, whereas the most important feature for the second component (Dim-2 or PC2) was the CD19^+^PD1^+^ level (Figure 3,C,D).

**Figure 4:**
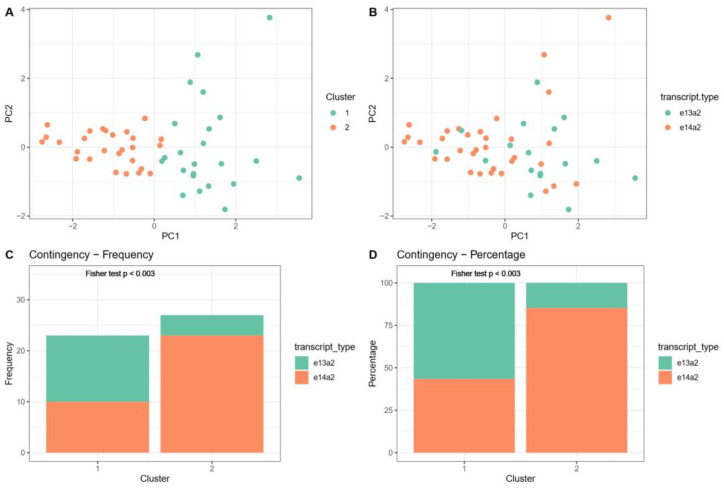
The outcomes of cluster analysis. (**A**) The dot plot from the principal component analysis (PCA) illustrates the scatter of data points along the first two principal components, PC1 and PC2. The dots are color-coded to represent two distinct clusters, visually distinguishing the data groups according to their PCA scores. (**B**) The PCA clustering plot reveals the dispersion of events along the first two principal components (PC1 and PC2), with colors indicating the different transcript types detected. (**C**) The bar plot from a contingency table details the distribution of patients categorized by PCA-derived clusters (marked as ‘Cluster’) across various transcript type sub-groups. The plot includes a *p* value from Fisher’s test, providing a measure of the statistical significance of the differences observed between clusters. (**D**) The bar plot, representing relative values from a contingency table, shows the distribution of patients within PCA-defined clusters (labeled as ‘Cluster’) across identified transcript type sub-groups. It also includes a *p* value from Fisher’s test, which helps to evaluate the statistical significance of the observed relationships between groups.

PCA separated the CML patients into two groups defined by k-means clustering, indicating significant heterogeneity within the studied population (Figure 4A,B). We found significant associations between obtained Cluster metrics (Cluster 1 and 2) and *BCR::ABL1* gene transcript types e13a2 or e14a2 (*p* = 0.003, 95% CI: 0.026 to 0.595, OR = 0.14, Fisher test), showing that the relative frequency of e13a2 is lower in patients grouped into Cluster 2 in comparison to those of Cluster 1, whereas the e14a2 transcript was more common for Cluster 2 patients in comparison to those of Cluster 1.

### 3.5. Immunological Characteristics of CML Patients with the e13a2 or e14a2 Transcripts After Imatinib Discontinuation

**Figure 5:**
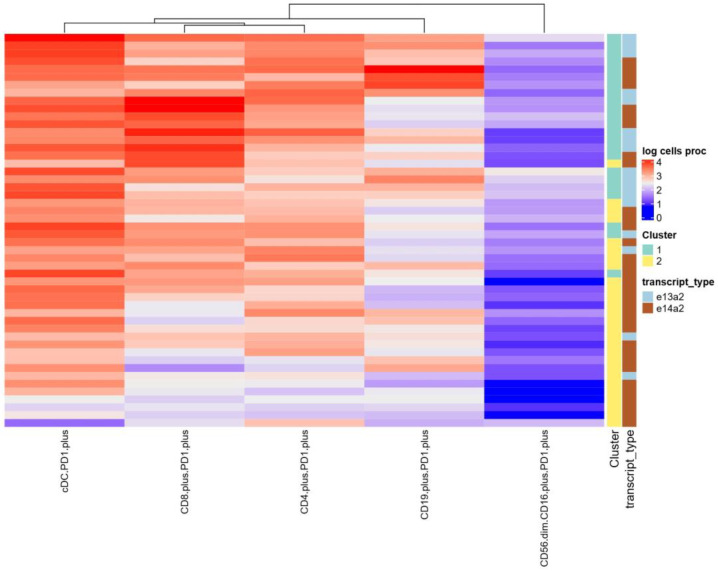
Heatmap of log-transformed percentages of selected immunological cell populations in patients. The heatmap displays patients in rows and selected immunological cell populations in columns, showing log-transformed percentages of cells. Two annotations are included: ‘Cluster,’ grouping patients based on previous PCA analysis results; and ‘transcript_type,’ indicating the presence of specific transcript types. Visualization aids in identifying patterns of immune cell distribution relative to patient PCA clusters and transcript types, highlighting underlying biological associations.

The heat map illustrates the logarithmic (log-normalized) number of immune cells and displays the patients in rows and the selected immune cell populations in columns, showing the log-transformed percentage of cells. The data were additionally grouped using the PCA method, which allowed for the separation of individual clusters. The map also includes transcript type annotations, allowing for a better understanding of differences in the percentages of immune populations between groups. The group of patients with the e13a2 subtype is predominantly located in Cluster 1 and exhibits the highest proportion of PD1-expressing cells (Figure 5).

**Figure 6:**
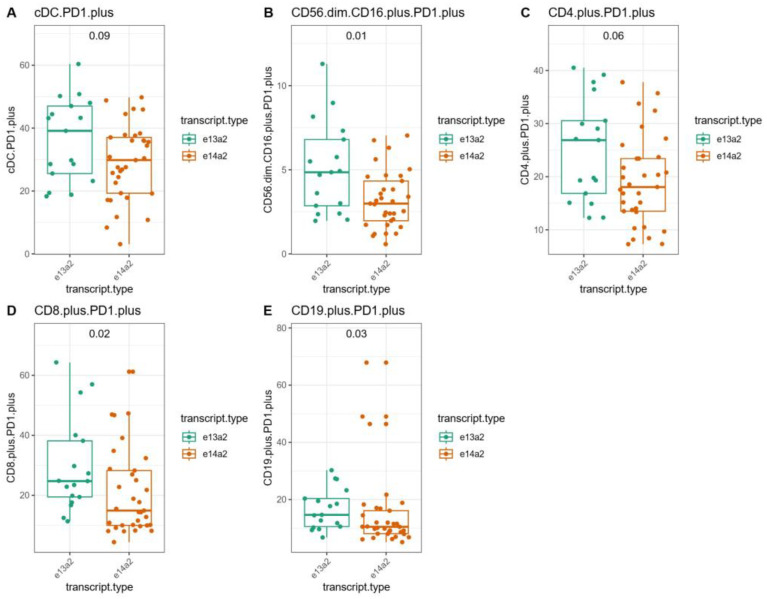
Box plots of immunological cell populations by transcript type with Mann–Whitney–Wilcoxon test results. The box plots depict the distribution of specific immunological cell populations among patients, grouped according to the type of transcript detected in each patient. The significance of differences between groups was evaluated using the Mann–Whitney–Wilcoxon test, with *p* values displayed here. (**A**) BDCA.1.plusPD1.plus (cDC.PD1.plus), (**B**) CD56.dim.CD16.plus.PD1.plus, (**C**) CD4.plus.PD1.plus, (**D**) CD8.plus.PD1.plus, and (**E**) CD19.plus.PD1.plus.

**Figure 7:**
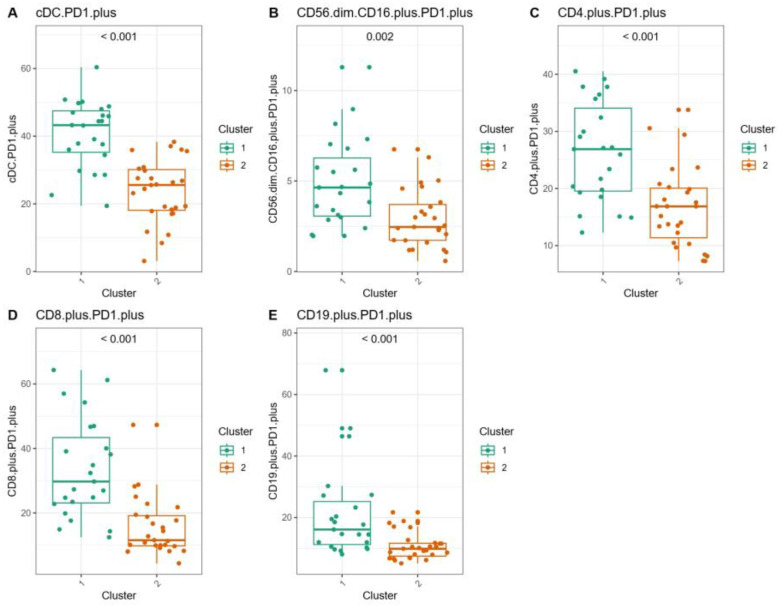
Box plots of immunological cell populations by PCA clusters with Mann–Whitney–Wilcoxon test results. The box plots depict the distribution of specific immunological cell populations among patients, grouped according to PCA clusters (presented in Figure 4A). The significance of differences between groups was evaluated using the Mann–Whitney–Wilcoxon test, with *p* values displayed here. (**A**) BDCA.1.plusPD1.plus (cDC.PD1.plus), (**B**) CD56.dim.CD16.plus.PD1.plus, (**C**) CD4.plus.PD1.plus, (**D**) CD8.plus.PD1.plus, and (**E**) CD19.plus.PD1.plus.

The box plots showed the distribution of the percentage of immunological cell populations among patients, grouped according to the type of *BCR::ABL1* gene transcript (Figure 6) and to PCA clusters (Figure 7). The significance of the differences between the groups was evaluated using the Mann–Whitney–Wilcoxon test. The patients with the e13a2 transcript had significantly higher percentages of CD56^dim^CD16^+^PD1^+^ cells (*p* = 0.01, median 4.85 vs. 2.99), CD8^+^PD1^+^ cells (*p* = 0.02, median 24.72 vs. 14.91), and CD19^+^PD1^+^ cells (*p* = 0.03, median 14.67 vs. 10.48) compared to the patients with the e14a2 transcript type. In the patients grouped in Cluster 1, selected based on the MCFS-ID algorithm, there was a significantly higher percentage of cells expressing PD1, cDC PD1^+^ cells (*p* < 0.0001, median 43.27 vs. 25.56), CD56^dim^CD16^+^PD1^+^ NK cells (*p* = 0.002, median 4.64 vs. 2.46), CD4^+^PD1^+^ cells (*p* < 0.001, median 26.89 vs. 16.86), CD8^+^PD1^+^ cells (*p* < 0.0001, median 29.74 vs. 11.56), and CD19^+^PD1^+^ cells (*p* < 0.001, median 16.12 vs. 9.87) compared to the patients in Cluster 2. The -log10 corrected *p* values obtained from the Mann–Whitney–Wilcoxon test comparing the percentages of specific immune cell populations across patient groups for the “transcript type” and “cluster” categories were further illustrated using a Manhattan plot (Supplementary Materials, Figures S2 and S3).

We found a positive correlation between the duration of DMR and the duration of imatinib treatment before the TFR trial in both the groups of patients expressing e13a2 (R = 0.87, 95% CI: 0.68 to 0.95, *p* < 0.0001) and e14a2 (R = 0.50, 95% CI: 0.18 to 0.72, *p* = 0.0028) transcripts (Supplementary Materials, Figure S4).

## 4. Discussion

Studies have demonstrated that patients with the e14a2 transcript respond more effectively to standard doses of imatinib, achieving lower *BCR::ABL1* transcript levels faster than those with the e13a2 transcript [27]. The presence of an additional 25 amino acids in the e14a2 transcript determines the changes in the structure of the BCR::ABL1 kinase binding domain [28], which may be associated with reduced tyrosine kinase activity and thus an enhanced response to TKIs. In contrast, the e13a2 transcript is often linked with higher tyrosine kinase activity, which may necessitate higher doses of TKIs or the use of second-generation TKIs to achieve similar molecular responses as those observed in patients with the e14a2 transcript [27].

It is known that CML is an immunologically active myeloproliferative neoplasm, primarily considering the remissions achieved after allogeneic hematopoietic stem cell transplants, infusions of donor lymphocytes or TKI treatment, which exhibit immunomodulatory properties. Therefore, immune surveillance of residual leukemic cells might be responsible for keeping patients in TFR. Accordingly, we attempted to investigate the differences in the immune system of patients with the most common transcript types. The observed increased expression of PD1 in immune cells, especially cytotoxic lymphocytes, may suggest weaker anti-tumor activity of the immune system in patients with the e13a2 subtype. The underlying immunological mechanisms contributing to these differences are complex and not fully understood. However, it is speculated that the presence of exon 14 in the e14a2 transcript might enhance its immunogenic properties, possibly by presenting unique epitopes that elicit a stronger immune response [29]. In contrast, the e13a2 transcript, lacking this exon, may not provoke as strong of an immune response, thereby requiring higher TKI doses to control the disease effectively [27]. The poorer outcomes for the e13a2 patients may be the result of impaired anti-tumor activity of the immune system. Studies show that higher numbers of lymphocytes expressing molecules such as PD1 or TIM3 are associated with molecular recurrences [12,15] and lower rates of achieving MMR or DMR [30]. Checkpoint molecule expression may be crucial in immunological mechanisms, and the poorer outcomes of the e13a2 patients may be related to PD1^+^ exhausted cells, as noted in this study. The increased number of PD1-expressing cells is associated with a higher likelihood of losing molecular remission after the discontinuation of imatinib, which suggests that immune checkpoint dynamics, such as PD1 molecule expression, may play a significant role in maintaining TFR [31]. This could suggest more robust immune surveillance in the e14a2 patients, potentially contributing to better control of residual disease and a greater likelihood of maintaining remission without the need for continuous treatment.

Studies have shown that patients with the e14a2 transcript often show not only better responses to TKIs, but also higher rates of profound molecular response compared to patients with the e13a2 transcript [20,32,33]. This might be important because a sustained deep molecular response is crucial to achieving TFR [2,34,35]. Moreover, in studies on the differences between patients with e13a2 and e14a2 transcripts, it was noted that patients with the e14a2 transcript had better rates of survival free from transformation to the blast phase and a greater chance of maintaining molecular remission at the MR4.5 level [18,21,34,36]. A meta-analysis by Chen et al. [37] confirmed these results, showing that the e14a2 transcript is associated with faster, deeper, and more sustained molecular responses, as well as better overall survival compared to e13a2. In contrast, some studies indicate that the type of *BCR::ABL1* transcript may not significantly impact overall survival or CML-related death [18,38]. However, the type of transcript is suggested to influence the speed and depth of the molecular response to TKI therapy, which indirectly affects TFR outcomes [27,39]. The lower response rates and greater need for aggressive TKI therapy in e13a2 patients imply a lower likelihood of achieving the deep molecular responses required for TFR. This necessitates more intensive monitoring and possibly extended therapy duration to improve their chances of maintaining remission without treatment [27]. However, one significant issue with the RT-qPCR method in quantifying the e13a2 transcript in CML patients is the potential for overestimation. This phenomenon can be attributed to the technical aspects of the RT-qPCR assay, such as primer efficiency and the secondary structures of the e13a2 transcript that can affect the accuracy of amplification. Differences in PCR amplification efficiency between the e13a2 and e14a2 transcripts result from the size of the amplicons, therefore, potentially overestimating the amount of the e13a2 transcript in the RT-qPCR method [40]. The overestimation of the e13a2 transcript can lead to an apparent higher burden of disease, which might not accurately reflect the true clinical status of the patient. Consequently, this can explain several clinical observations, such as poorer results of TFR trials or a lower rate of DMR in response to TKI treatment in patients with the e13a2 transcript. Nevertheless, these differences are small considering the variability of the test results and do not affect overall survival; however, the technical limitations of RT-qPCR should be considered [41,42]. Addressing these technical limitations is essential to ensure the accurate measurement of *BCR::ABL1* levels and improve the management of CML patients with the e13a2 transcript. Consideration should be given to optimizing the assay conditions, using more specific primers, and validating the results with alternative quantification methods to ensure the accurate measurement of *BCR::ABL1* levels or using methods such as ddPCR, used in this study, which is insensitive to differences in amplification kinetics and allows for more accurate discrimination between the two types of transcripts with less variable results.

In summary, our results provide additional arguments supporting the thesis about the biological differences characterizing CML with different types of *BCR::ABL1* gene transcripts and suggest that the *BCR::ABL1* transcript type may play a critical role in the immunological and clinical outcomes of CML. Patients with e13a2 transcripts, due to their higher kinase activity and lower response rates, face greater challenges in achieving TFR and may require more intensive therapeutic strategies. Here, we provide an immunological explanation in the context of higher PD1 expression in the immune cells of patients with the e13a2 transcript. Understanding these differences might be crucial for optimizing TFR strategies toward improving the long-term outcomes for CML patients.

## Figures and Tables

**Table 1:** Characteristics of CML patients enrolled in this study.

	e14a2	e13a2	*p* Value
Number of patients, N (%)	33 (64.7)	18 (35.3)	
Gender, Male, N (%)	16 (48.5)	6 (33.3)	0.39
Age, years median (95% CI)	65; 59–74	59.5; 46–76	0.74
ELTS score, N (%)			0.095
low	23 (69.7)	9 (50.0)	
intermediate	5 (15.2)	2 (11.1)	
high	0 (0.0)	2 (11.1)	
EUTOS score, N (%)			0.19
low	26 (78.8)	13 (72.2)	
intermediate	4 (12.1)	0 (0.0)	
high	3 (9.1)	4 (22.2)	
Sokal score, N (%)			0.69
low	26 (78.8)	13 (72.2)	
intermediate	4 (12.1)	2 (11.1)	
high	3 (9.1)	3 (16.7)	
Hasford score, N (%)			0.19
low	24 (72.7)	10 (55.6)	
intermediate	7 (21.2)	3 (16.7)	
high	2 (6.1)	4 (22.2)	
Neutropenia, N (%)			0.86
1/2	4 (12.1)	2 (11.1)	
2	0 (0.0)	0 (0.0)	
3	1(3.0)	1 (5.6)	
3/4	2 (6.1)	2 (11.1)	
Anemia, N (%)			0.47
1/2	14 (42.4)	4 (22.2)	
2	0 (0.0)	1 (5.6)	
3	0 (0.0)	0 (0.0)	
3/4	0 (0.0)	0 (0.0)	
Thrombocytopenia, N (%)			0.80
1/2	4 (12.1)	2 (11.1)	
2	0 (0.0)	0 (0.0)	
3	0 (0.0)	1 (5.6)	
3/4	1 (3.0)	1 (5.6)	
Duration of DMR ^1^, months median (95% CI)	83; 74–100	74.5; 51–91	0.22
Duration of TKI treatment ^1^, months median (95% CI)	116; 102–124	103.5; 61–128	0.16
Lost MMR ^2^, N (%)	8 (24.2)	4 (22.2)	0.68
Lost DMR ^2^, N (%)	11 (33.3)	9 (50.0)	0.42

Abbreviations: DMR, deep molecular response; ELTS score, EUTOS Long-Term Survival score; MMR, major molecular response. Categorical variables were assessed using the chi-square test (χ^2^) or Fisher’s exact test, based on the size of the subgroups, while continuous variables were analyzed using the Mann–Whitney test (U-test). Statistically significant findings were those with an adjusted *p* value below 0.05. ^1^ before TFR ^2^ at 3 months of TFR.

## Data Availability

The data that support the findings of our study are available upon request from the corresponding author.

## Supplementary Materials

The following supporting information can be downloaded at: https://www.mdpi.com/article/10.3390/jcm14072304/s1, Figure S1: Principal features at the forefront of MCFS-ID ranking analysis for patients with transcript types e13a2 and e14a2; Figure S2: Comparative analysis of immunological cell populations across different transcript types; Figure S3: Comparative analysis of immunological cell populations across PCA clustering; Figure S4: Correlation between duration of DMR and duration of imatinib treatment before the TFR trial in patients with e13a2 (A) and e14a2 (B) transcripts.

## Abbreviations

The following abbreviations are used in this manuscript:

CML	Chronic Myeloid Leukemia
DC	Dendritic Cells
DMR	Deep Molecular Response
MCFS-ID	Monte Carlo Feature Selection and Interdependency Discovery
MMR	Major Molecular Response
NK	Natural Killers
NKT	Natural Killer T cells
PCA	Principal Component Analysis
PBMC	Peripheral Blood Mononuclear Cells
TFR	Treatment-Free Remission
TKI	Tyrosine Kinase Inhibitor
TREG	Regulatory T Lymphocytes
