# Supplementary material for: The PD1 Molecule May Contribute to Lower Treatment-Free Remission Rates in Patients with Chronic Myeloid Leukemia with the e13a2 Transcript
Source: J Clin Med. 2025 Mar 27;14(7):2304. doi: 10.3390/jcm14072304 (PMC11989261; doi:10.3390/jcm14072304)
Supplement: Supplementary file 1 [file jcm-14-02304-s001.zip › jcm-3517314-supplementary/Supplementary Figures.pdf]

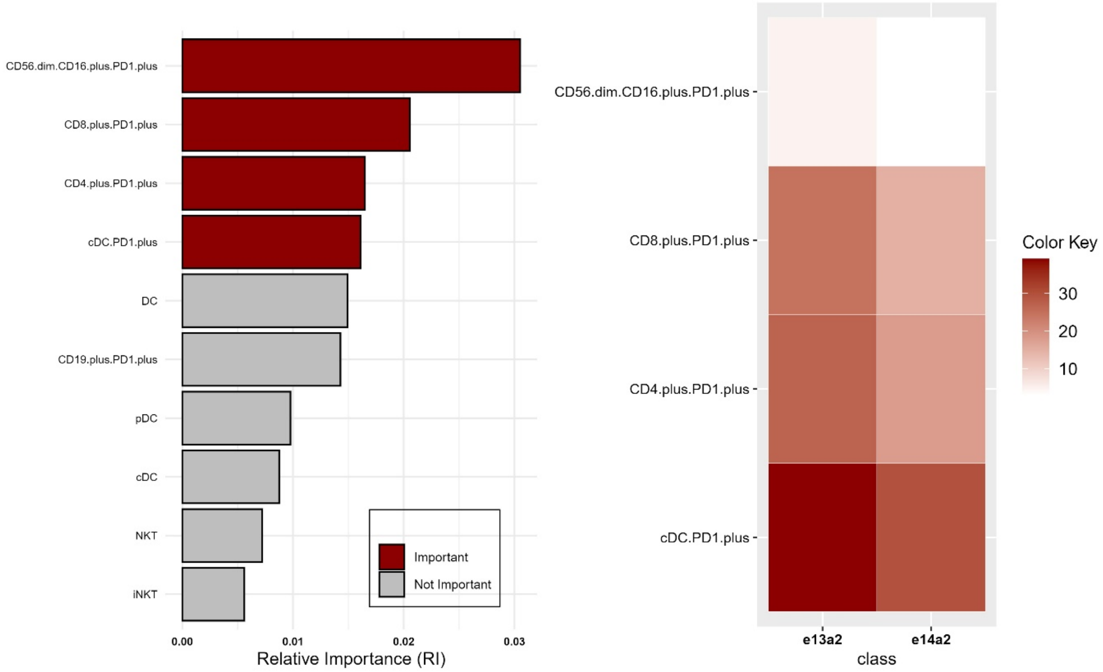

Supplementary Figure S1. Principal features at the forefront of MCFS-ID ranking analysis for patients with transcript types e13a2 and e14a2. A) The plot shows the Relative Importance (RI) of top-ranked features, using red to highlight significant features above the cutoff point and gray for those below it (for detailed criteria, refer to the Methods section). B) Thr plot presents the average values of significant features for each decision class aligned with the analyzed clinical grouping variable.

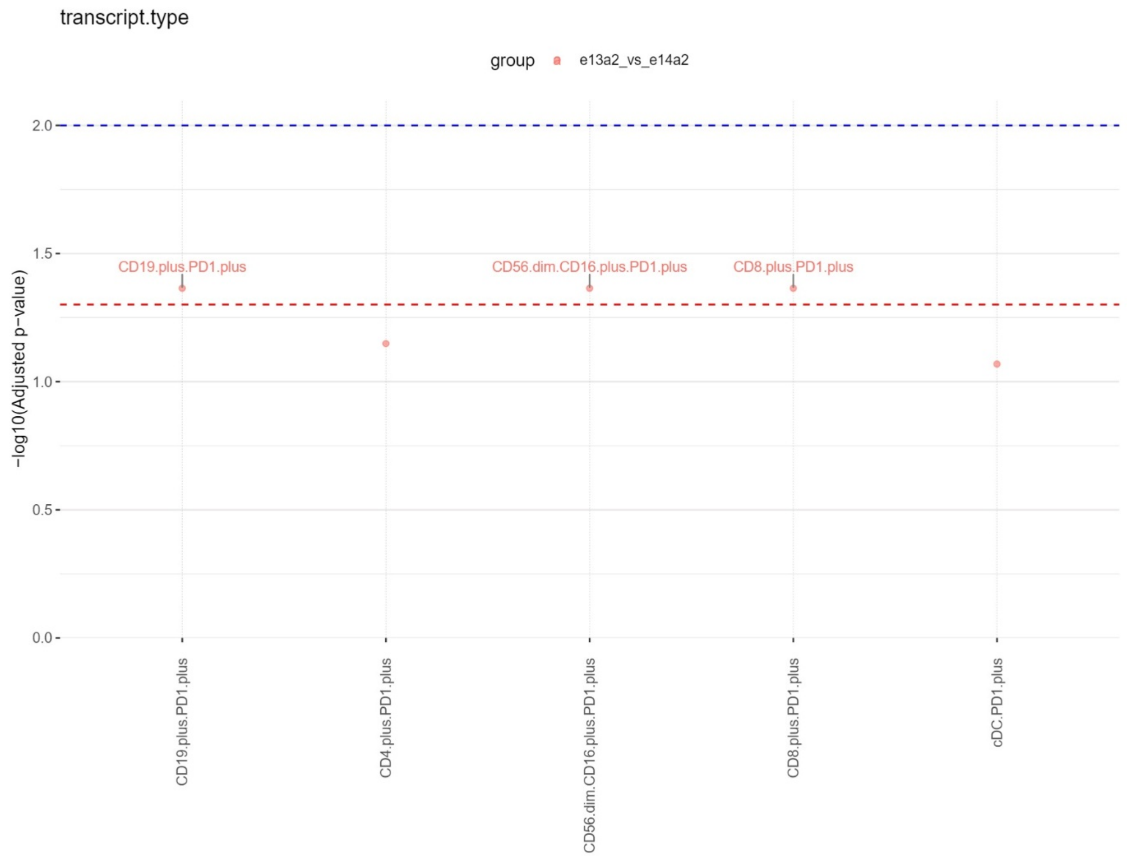

Supplementary Figure S2. Comparative analysis of immunological cell populations across different transcript types. The Manhattan plot displays the -log10 adjusted *P* values derived from the Mann-Whitney-Wilcoxon test, comparing the percentages of specific immune cell populations among patient groups. Thresholds for significance are marked by dashed red lines for *P* values at 0.05 and blue lines for 0.01. Points indicating statistically significant differences are annotated directly on the plot for clear visualization.

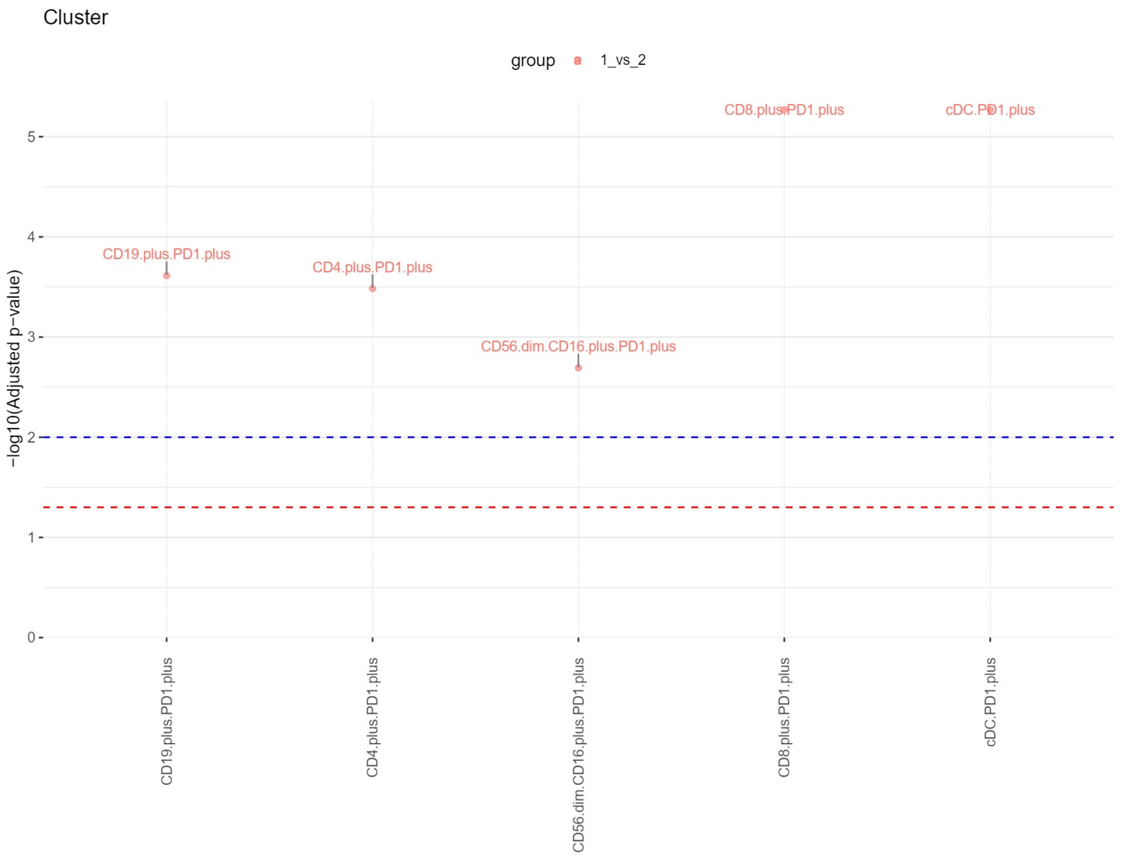

Supplementary Figure S3. Comparative analysis of immunological cell populations across PCA clustering. The Manhattan plot displays the -log10 adjusted *P* values derived from the Mann-Whitney-Wilcoxon test, comparing the percentages of specific immune cell populations among patient groups. Thresholds for significance are marked by dashed red lines for *P* values at 0.05 and blue lines for 0.01. Points indicating statistically significant differences are annotated directly on the plot for clear visualization.

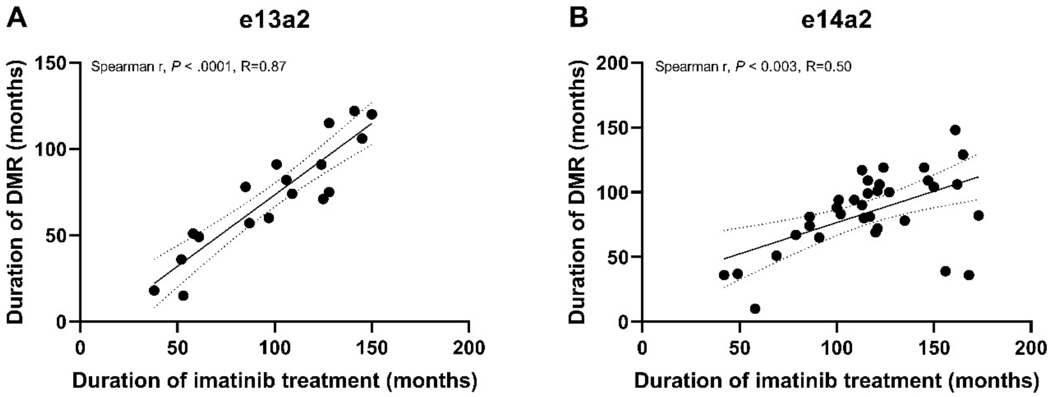

Supplementary Figure S4. Correlation between duration of DMR and duration of imatinib treatment before TFR trial in patients with e13a2 (A) and e14a2 (B). The duration of DMR is directly related to the duration of imatinib treatment in patients with the e13a2 (R=0.87, 95% CI: 0.68 to 0.95, $P < .0001$) and e14a2 (R=0.50, 95% CI: 0.18 to 0.72, $P = 0.0028$) transcript (Spearman r).
